# Equine-assisted services for people living with dementia: a systematic review

**DOI:** 10.1186/s13195-024-01453-4

**Published:** 2024-04-09

**Authors:** Menka Sebalj, Ali Lakhani, Andrea Grindrod, Rwth Stuckey

**Affiliations:** 1 School of Psychology and Public Health, La Trobe University, Kingsbury Drive, Bundoora, Vic 3086 Australia; 2 The Hopkins Centre, Menzies Health Institute Queensland, Griffith University, Logan Campus, University Drive, Meadowbrook, QLD 4131 Australia; 3 Palliative Care Department, Eastern Health, 251 Mountain Highway, Wantirna, VIC 3152 Australia

**Keywords:** Dementia, Equine-assisted services, Health and wellbeing, Systematic review

## Abstract

**Background:**

Dementia has a significant impact on the social, physical, and psychological wellbeing of people living with dementia, their families and society. Animal-assisted interventions can have positive effects on the health and wellbeing of people living with dementia. Equine-assisted services are animal-assisted non-pharmacological interventions which have improved the health and wellbeing of diverse populations. The impact of participating in equine-assisted services on the health and wellbeing of people with dementia is unclear. A systematic review was conducted to synthesise evidence investigating the effects of participating in equine-assisted services on the health and wellbeing of people living with dementia.

**Design:**

Systematic review following the Preferred Reporting Items for Systematic Reviews and Meta-Analyses (PRISMA) guidelines.

**Methods:**

The databases CINAHL, EMBASE, MEDLINE, and Web of Science were searched for any research published prior to 14 June 2023. Peer-reviewed publications in the English language utilizing methods deriving quantitative and/or qualitative data were eligible. Methodological quality of included studies was assessed using the Mixed Methods Appraisal Tool. Findings from studies were synthesised using a deductive approach.

**Results:**

Of the 223 articles screened, six met the inclusion criteria: four quantitative and two qualitative studies. The six studies represent four separate equine interventions. Studies were of moderate to strong quality. Participants were people living with dementia (*n* = 44, mean age range 70–83 years), dementia care partners (*n* = 5, mean age 58), and equine-assisted services providers (*n* = 5). Interventions varied in duration, activities conducted, outcomes measured, and measurement tools used. Studies found a favourable impact of participating in equine-assisted services on the neuropsychiatric symptoms and quality of life of people living with dementia. Participating in equine-assisted services improved well-being, functional abilities, social participation, and communication, while also having a positive effect on social, emotional, and behavioural outcomes, and physical health.

**Conclusions:**

The limited but high-quality literature investigating the impact of equine-assisted services among people living with dementia suggests that equine-assisted services can have a positive impact on the health and wellbeing of people living with dementia. Additional robust studies contributing to the evidence base are warranted; such studies can support the development of programs and further elucidate the impact of participation.

## Introduction

Dementia is an umbrella term for a collection of conditions that affect cognitive function [1, 2]. Types of dementia include vascular dementia, Lewy body disease, and frontotemporal dementia. Alzheimer’s disease, accounts for approximately 70% of dementia cases [1, 2]. Approximately 55 million people worldwide live with dementia, and with an ageing population, by 2050, this number is expected to increase to 152 million [1, 3]. Dementia is a leading cause of mortality, and in 2016 accounted for 2.4 million deaths [3, 4].

Dementia affects memory, thinking, orientation, comprehension, judgment, and behaviour. Specific symptoms include memory loss, confusion, forgetfulness, and changes in mood, behaviour, and/or personality [2, 5], which can affect a person’s normal social or working life and activities of daily living [1, 2, 5]. Neuropsychiatric symptoms of dementia include certain behaviours (agitation, aggression, resistance to care, restlessness, irritability, confusion), and psychological manifestations (psychosis, delusions, hallucinations, depression, anxiety, apathy) [1, 4, 6].

### Management of dementia symptoms

The causes of dementia are not always known and there is no cure. Consequently, dementia care focuses on managing neuropsychiatric symptoms, and providing support to promote quality of life (QoL) and activities of daily living for people living with dementia [4, 5, 7]. Interventions that assist in neuropsychiatric symptom management for people living with dementia can be pharmacological and non-pharmacological and include medication, environmental changes, and social, physical, and/or psychological support [1]. Considering that research has established that pharmacological treatment can have limited or no efficacy and is sometimes accompanied by severe side-effects [1, 2, 6], non-pharmacological interventions are encouraged as an initial ‘safer’ alternative to pharmacological approaches [8].

Animal-assisted interventions are a non-pharmacological approach to dementia care. Animal-assisted interventions generally involve human-animal interactions to achieve health and wellbeing benefits in humans [9]. Animal-assisted interventions can involve dogs, horses, fish, cats, donkeys, and guinea pigs [10, 11]. There is a growing body of evidence investigating the efficacy of Animal-assisted interventions to treat the neuropsychiatric symptoms of dementia [12, 13]. For people living with dementia, Animal-assisted interventions can improve QoL [11, 12], reduce depression levels [14], reduce agitation and aggression [11], reduce neuropsychiatric symptoms of dementia [15], and improve social behaviours [11].

### The value of equine-assisted services)

Equine-assisted services fit within the umbrella of animal-assisted interventions. Equine-assisted services can be educative, recreational, and/or therapeutic. They often involve interactions with horses (and other equines such as donkeys or mules) [16] to improve human health and wellbeing [17]. There are a diversity of definitions and terminologies used to describe equine-assisted services [18]. This is further complicated by the large range of equine-assisted services available and the different providers of these services [19]. To address this issue, Wood and colleagues [19] developed a consensus document for recommendations of terminology describing the different services that incorporate equines. They distinguished 12 types of equine services, categorized into three areas of professional work: therapy, learning and horsemanship [19]. Equine-assisted services was proposed as the umbrella term for all the equine services in these areas that are used by professionals to benefit humans [19]. Thus, the term equine-assisted services is used throughout this review with reference to any equine intervention or service used.

Reviews investigating the efficacy of equine-assisted services confirm that participants receive benefit, however results are mixed. For example, an umbrella review of equine-assisted services found that, for children with cerebral palsy, engagement in equine-assisted services can produce either significant benefits or none [18]. These benefits include improvements in social behaviour and engagement [20], a reduction in depression and challenging behaviours in children [20], improvement in physical health [20], and significant improvements in measured outcomes for children with autism. However, reviews of equine-assisted services indicate methodological flaws and weaknesses in the evidence base. These include few studies in the area, studies including small sample sizes, few high-quality studies, the presence of a risk of bias in most studies, and finally, a diversity of study designs, outcomes and measurement tools used, making it difficult to compare studies and limiting generalisability [11, 14, 15, 21].

The few reviews investigating the impact of animal-assisted interventions for people living with dementia have generally considered how assistance dogs can be used to promote health among people living with dementia [11, 14, 15, 21]. These reviews are valuable and have confirmed that animal-assisted interventions may reduce depression and improve behavioural symptoms [14, 15], however they also find that animal-assisted interventions do not have a significant impact on cognitive, QoL, and activity of daily living outcomes [14, 15]. While valuable, these reviews have not included terms specific to equine-assisted services (the reviews have included terms broadly relating to animal-assisted interventions ), and thus, the impact of participating in equine-assisted services for people living with dementia is unclear.

Given the success that equine-assisted services have had in addressing QoL outcomes for diverse groups, there is potential that equine-assisted services may produce unique benefits for people living with dementia, beyond those identified across a broad range of animal-assisted interventions. An evidence-based review is necessary to establish the state of knowledge in the area. This systematic review aims to establish the extent of studies investigating the effects of participating in equine-assisted services on people living with dementia. Research questions include:

What is the impact of participating in equine-assisted services on the health and wellbeing of people living with dementia?

What outcomes have studies investigating the impact of equine-assisted services on people living with dementia addressed?

## Methods

### Study design

A systematic review is one of 14 types of reviews intended to inform evidence-based practice [22]. A systematic review methodology [22] was chosen in-line with this study’s aim to systematically search, appraise, and synthesise the evidence of equine-assisted services for improving the health and well-being of people living with dementia. This systematic review followed the Preferred Reporting Items for Systematic Reviews and Meta-Analyses (PRISMA) framework [23]. The review was registered with Prospero (ID CRD42022350706).

### Search strategy

The following databases were searched via the [blinded for review] University online library. Research published up until 14 June 2023 from the following databases were considered: CINAHL, EMBASE, MEDLINE, Web of Science. The following string was used during a title search of each database: (cognitive OR delirium OR “chronic confusion” OR dementia* OR Alzheimer*) AND (“equine-assisted service*” OR “equine-facilitated learning” OR “equine-assisted therap*” OR “equine-assisted intervention*” OR “equine-facilitated psychotherapy” OR EFP OR “horse* riding” OR horse* OR hippotherapy OR “therapeutic horse riding” OR “equine therapy” OR equine OR “riding”). Additionally, the search terms were entered into Google Scholar.

### Eligibility criteria

The inclusion criteria for studies in this review were: publication in a peer-reviewed journal; written in the English language; a primary research article; include an equine-assisted service (incorporating horses or other equines); involve people living with dementia as the primary group, and where multiple groups were included provided findings for people living with dementia alone. The exclusion criteria for this review were: virtual interventions or interventions not using live animals (e.g., soft toys and simulations); studies which were not dementia specific; audit reports, poster abstracts, protocols, informative pieces of writing; publications in a non-peer reviewed journal; and secondary research articles and/or reviews.

### Screening and study selection

The results of the database searches were deposited into a reference management software EndNote [24] and all duplicates were removed. One researcher (AL) reviewed the titles and abstracts of the studies against the eligibility criteria. The selected full-text articles were assessed for eligibility by two researchers (AL and MS) and the reference lists of the articles scanned for further pertinent literature. Where there was doubt about the eligibility of a source throughout the screening process, it was discussed by both AL and MS until consensus was reached.

### Data extraction and synthesis

Data extraction was undertaken by MS and AL, and differences in opinion were resolved with discussion between the two researchers. Data extracted from the included studies was entered into a table in Microsoft Word. The data extracted included: study reference and country, type of equine-assisted services provided, participant information (sample number, age and gender), and study design. Data on intervention settings, animal characteristics, and risks associated with equine-assisted services was also obtained. A deductive synthesis approach was utilized, and findings were summarised in line with the following domains: characteristics of the equine-assisted service, study outcomes, and study findings.

### Quality appraisal

Methodological quality of included studies was assessed using the Mixed Methods Appraisal Tool (MMAT), version 2018 [25]. The MMAT is useful tool when appraising different study designs investigating a topic of interest for a systematic review and can used for qualitative, quantitative and mixed-method studies [25]. One researcher administered the MMAT (MS) and a second researcher (AL) reviewed the appraisal. Differences in quality ratings were resolved with discussion between the researchers until consensus was reached. Each criterion was designated 1 if it met the methodological criterion according to the MMAT, or 0 if it did not meet the quality of the criterion or was unclear.

## Results

**Fig. 1:**
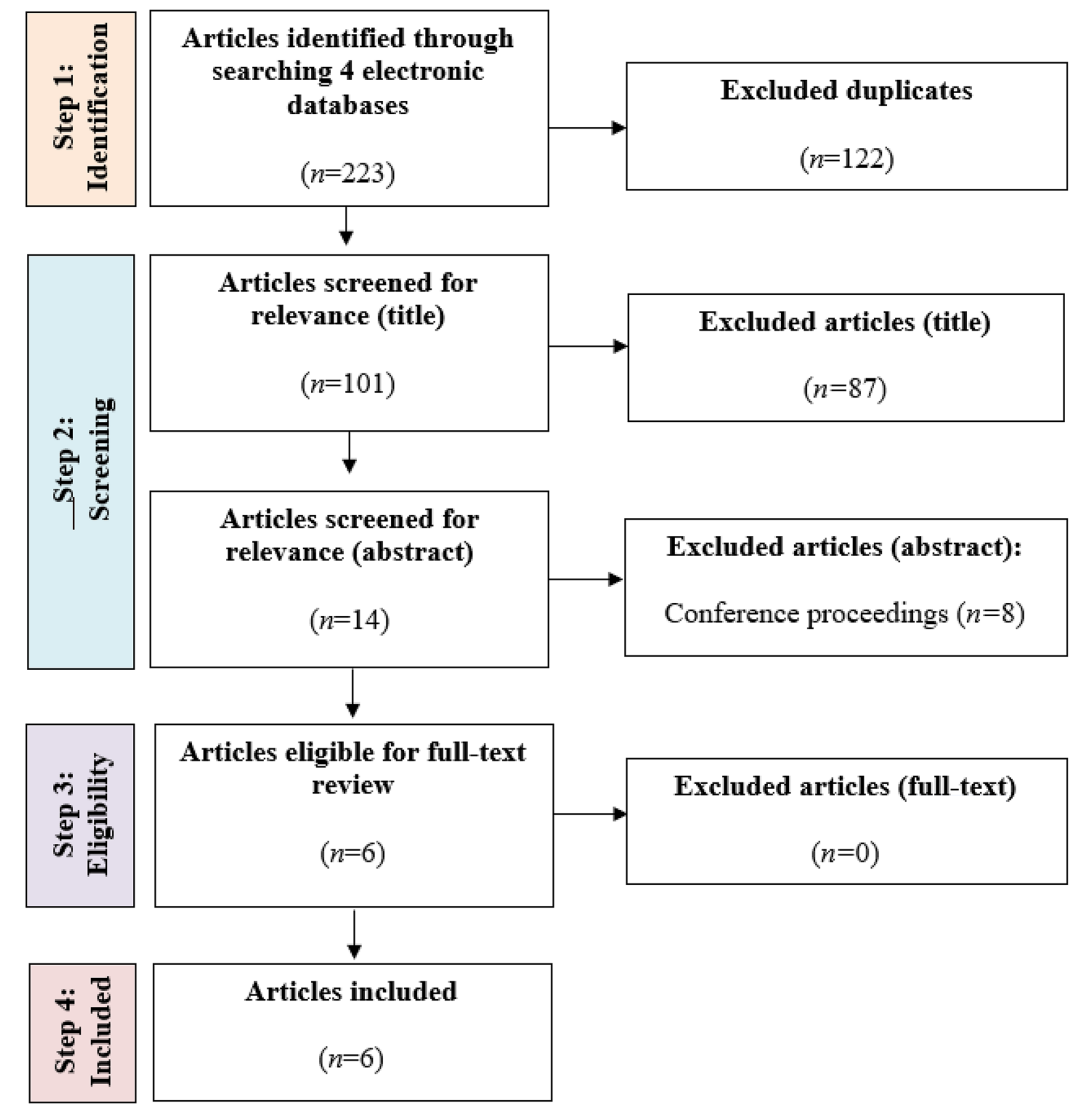
PRISMA diagram of the study selection process

A total of six publications were included in this review. The study selection process is shown in Fig. 1. The six publications are underpinned by the following intervention types: two equine assisted therapy interventions [26, 27], one equine-assisted activities program intervention [28, 29], and one adaptive riding intervention [30, 31]. Of these six publications, two relate to the same study [28, 29].

### Quality assessment

The results of the methodological quality assessment of the included publications are reported in Table 1. Four publications were of medium to high quality [26–28, 31] and two were high quality [29, 30].

**Table 1:** Quality appraisal of the included studies using the Mixed Methods Appraisal Tool (MMAT)

Mixed Methods Appraisal Tool (MMAT), v 2018		Study					
Category of study designs	**†Methodological quality criteria**	**Borges de Araujo et al. (2018)** [26]	**Dabelko-Schoeny et al. (2014)** [27]	**Fields et al. (2018)** [28]	**Fields et al. (2019)** [29]	**Lassell et al. (2021)** [31]	**Lassell et al. (2022)** [30]
Screening Questions	**S1.** Are there clear research questions?	0	0	1	1	1	1
	**S2.** Do the collected data allow to address the research questions?	1	1	1	1	1	1
1. Qualitative study criteria	**1.1.** Is the qualitative approach appropriate to answer the research question?				1		1
	**1.2.** Are the qualitative data collection methods adequate to address the research question?				1		1
	**1.3.** Are the findings adequately derived from the data?				1		1
	**1.4.** Is the interpretation of results sufficiently substantiated by data?				1		1
	**1.5.** Is there coherence between qualitative data sources, collection, analysis and interpretation?				1		1
3. Quantitative non-randomized study criteria	**3.1.** Are the participants representative of the target population?	1	1				
	**3.2.** Are measurements appropriate regarding both the outcome and intervention (or exposure)?	1	1				
	**3.3.** Are there complete outcome data?	1	1				
	**3.4.** Are the confounders accounted for in the design and analysis?	0	1				
	**3.5.** During the study period, is the intervention administered (or exposure occurred) as intended?	1	1				
4. Quantitative descriptive	**4.1.** Is the sampling strategy relevant to address the research question?			1		1	
	**4.2.** Is the sample representative of the target population?			0		0	
	**4.3.** Are the measurements appropriate?			1		1	
	**4.4.** Is the risk of nonresponse bias low?			0		0	
	**4.5.** Is the statistical analysis appropriate to answer the research question?			1		1	
‡Quality Rating		5	5	5	6	5	7

†Quality criteria: 1 = criterion was met; 0 = criterion was not met or unclear

‡Quality rating (maximum rating is 7): 0 or 1 = low quality; 2 or 3 = low to medium quality; 4 or 5 = medium to high quality; 6 or 7 = high quality

Note: a blank space means a score is not applicable

### Characteristics of studies

Study characteristics are provided in Table 2. Five studies were from the USA [27–31] and one from Brazil [26]. All the included studies were published between 2018 and 2022, except for one study which was published in 2014 [27]. In relation to study design, four were quantitative [26–28, 31] and two were qualitative [29, 30]. Borges de Araujo et al. [26] utilized a quasi-experimental, uncontrolled pre-post design, whilst Fields et al. published quantitative [28] and qualitative [29] data from a mixed-methods case-study. The remaining studies utilised a randomised pre-post crossover study [27], a descriptive case study [31], and a qualitative case study design [30]. Sample sizes were small and ranged from 5 participants [29, 30] to 16 participants [27]. Non-random convenience sampling was used for all studies.

**Table 2:** Characteristics of the studies included in this systematic review

Study reference	Equine-assisted service or program	Participants (*n*, x̄ age, % female)	Study design	Characteristics of Intervention	Outcomes	Finding(s)
Borges de Araujo et al. (2018) [26] Brazil	Equine-Assisted Therapy with docile horses exclusively used for equine therapy	People diagnosed with mild to moderate Alzheimer’s Disease (*n* = 9, 78.6, 33%)	Quasi-experimental, uncontrolled pre-post	20 sessions of Equine-Assisted Therapy, twice a week for 10 weeks with each session lasting 30 min	1.Balance (force plate) 2.Functional capacity (Timed Up & Go test & 30-sec chair stand test) 3.Cognition (Verbal fluency & Mini-Mental State Examination)	Improvement pre & post intervention for outcomes 1 & 2. No deterioration in cognitive function.
Dabelko-Schoeny et al. (2014) [27] USA	Equine-Assisted Therapy with 4 therapy horses	People diagnosed with early to moderate stage Alzheimer’s Disease or related dementia (*n* = 16, 78.1, 56%)	Randomised pre-post crossover	Weekly sessions, once a week for 4 weeks with 3 × 15-minute activities per session. Activities included: grooming, observation or interaction, painting	1.Behaviour & affect (Philadelphia Geriatric Center Affect Rating Scale) 2.Stress (Salivary cortisol concentrations) 3.Disruptive Behaviours (Nursing Home Behaviour Problem Scale)	Participants showed positive engagement with the activities. There was a reduction in behavioural problems post-intervention compared to the comparison group.
Fields et al. (2018) [28] USA	Equine-Assisted Activities Program with 3 trained horses and 1 pony at an accredited therapeutic riding centre	Residents in a long-term care facility with mild to moderate dementia (*n* = 6, 83.3, 67%)	Quantitative component of a mixed methods case study, comparing Equine-Assisted Activities Program to long-term care facility activities.	Weekly one-hour session of Equine-Assisted Activities Program for 8 weeks of riding, grooming, petting or observing horses. Long-term care facility activities included downtime, TV, meals & snacks, physical therapy, games, joke & riddle time	Quality of Life (QoL) indicators related to: 1.Time use (gaze, position & movement, conversation, participation) 2.Emotional wellbeing (apparent affect & agitation)	The QoL subdomains of conversation and apparent affect (pleasure) were more frequently observed during the Equine-Assisted Activities Program than long-term care facility activities. There was no difference between Equine-Assisted Activities Program and long-term care facility activities for gaze, position & movement, and agitation.
Fields et al. (2019) [29] USA	Equine-Assisted Activities Program, with 3 trained horses and 1 pony at an accredited therapeutic riding centre	Providers of the Equine-Assisted Activities Program (*n* = 5)	Qualitative component of a mixed methods case study from the providers perspective	Same as in Fields et al., 2018	Providers perceptions of Equine-Assisted Activities Program that relate to: 1.safety 2.QoL outcomes 3.acceptiblilty	Equine-Assisted Activities Program was found to be safe and acceptable by providers and contributed to positive QoL outcomes for participants.
Lassell et al. (2021) [31] USA	1.Adaptive Riding with 3 horses and 1 donkey at an accredited therapeutic riding centre 2.Adaptive Gardening	People diagnosed with dementia (*n* = 8, 74, 75%)	Descriptive case study	Two interventions of Adaptive Riding (*n* = 4) and Adaptive Gardening (*n* = 4), with a weekly one-hour session for 8 weeks	QoL indicators of: 1.Participation (gaze, communication, and active participation) 2.Emotional wellbeing (apparent affect & agitation)	Positive outcomes for QoL indicators for Adaptive Riding and Adaptive Gardening. Adaptive Riding participants engaged in more complex active participation compared to Adaptive Gardening participants.
Lassell et al. (2022) [30] USA	Adaptive Riding: Riding in the Moment, with Horses and donkeys at an accredited therapeutic riding center	People diagnosed with dementia (*n* = 5, 70, 80%) and dementia care partners (*n* = 5, 58, 70%)	Qualitative case study design	Weekly one-hour sessions of Riding in the Moment for 8 weeks with opportunity to groom, observe, pet and ride a horse or donkey	1.Well-being 2.Meaning through social connections 3.Function in daily life.	The program was deemed appropriate for people living with dementia and care partners. Care partners reported improved well-being and function in daily life for people living with dementia. Meaning through social connections improved for people living with dementia and carers.

Abbreviations: n = number of participants; $\overline {\rm{X}}$ = mean; QoL = Quality of Life.

### Participant characteristics

Participants from four studies were people diagnosed with mild to moderate dementia or Alzheimer’s disease and were recruited from an adult day services centre [27], a long-term care facility [28], unspecified local organisations [31], and a local referral centre for people living with dementia [26]. One study explored the impact of an equine-assisted intervention from the providers perspective [29] and another from the dementia care partners perspective [30]. The mean age of participants living with dementia ranged from 70 years [30] to 83.3 [28]. The mean age for dementia care partners was 58 years [30].

### Characteristics of equine-assisted services

The equine-assisted services examined in this review used horses [26–31], donkeys [30, 31] and a pony [28, 29]. Four studies conducted the equine-assisted service at an accredited therapeutic riding centre (Professional Association of Therapeutic Horsemanship International), and two studies used therapy horses that had docile temperaments and were used exclusively as therapy animals [26], and in therapeutic riding programs for children and teenagers diagnosed with autism and mental health conditions [27]. Only one study provided the characteristics of the horses used in their study [27].

The duration of equine-assisted services varied for each study: the longest consisted of 20 half-hour sessions of equine-assisted therapy, twice a week for 10 weeks [26]. The shortest comprised one session a week, of three 15-minute activities per session, for four weeks [27]. Two interventions consisted of weekly one-hour sessions for eight weeks [28–31]. The intervention activities were varied: one intervention encompassed horse riding [26], two offered a choice of riding or ground activities (grooming, petting, and observing) [28, 30, 31], and one was a non-riding intervention offering ground activities of grooming, observation, horse care, horse exercise, and horse painting [27]. Lassell et al. [31] was the only study to investigate the impact of two different interventions (adaptive riding and adaptive gardening). Fields et al. [28] and Fields et al. [29] investigated the impact of an Equine-Assisted Activities Program, compared to the control of participating in long-term care regular activities of downtime, snack and mealtime, TV, games, physical therapy, and joke/riddle time. Similarly, a crossover design was utilised to establish the impact of participating in an equine-assisted therapy intervention, compared to the control of participating in regular adult day service activities [27].

### Outcomes

Two interventions assessed cognitive and behavioural outcomes through the QoL indicators of time use and participation (gaze, position and movement, communication, and active participation) and emotional well-being (apparent affect and agitation) [28, 31]. Both studies used the *Activity in Context and Time* [28, 31] to measure outcomes. The *Activity in Context and Time* is a quantitative, computer-assisted tool that measures the activities, time-use patterns, and QoL indicators of people living with dementia in aged-care facilities or other care institutions [32].

Dabelko-Schoeny et al. [27] investigated the impact of participating in equine-assisted therapy on physiological and behavioural outcomes. The physiological outcome of stress was measured via salivary cortisol concentrations of participants, pre- and post-intervention. Behavioural outcomes were assessed using [1] a modified version of the Nursing Home Behaviour Problem Scale [33] to measure disruptive behaviour, and [2] the Philadelphia Geriatric Center Affect Rating Scale [34] to measure behaviour and affect [27]. The Nursing Home Behaviour Problem Scale was developed by Ray et al. [33] and is a 5-point Likert-type scale of 29 items representing common observable disruptive behaviours demonstrated by people living with dementia in nursing homes [33]. The Nursing Home Behaviour Problem Scale used in the study by Dabelko-Schoeny et al. [27] was reduced to 22-items and modified to included questions pertinent to the study population from an adult day centre [27]. Affect was measured using the Philadelphia Geriatric Center Affect Rating Scale, developed by Lawton et al. [34], and recorded observed expressions of sadness, anger, pleasure, anxiety/fear, or interest [27, 34].

Only one study [26] examined physical outcomes, through the assessment of balance (force plate) and functional mobility of participants (Timed Up and Go test and 30-second chair stand test). Balance was measured using the AccuSway Plus force plate from Advanced Mechanical Technology and Balance Clinic software; data was collected according to Mann et al. [35] and Teixeira et al. [36]. The Timed Up and Go test is used to identify elderly people at risk of falls [37] and measures the time taken to stand up from a chair, walk three metres, and return to sit in the same chair [26]. The 30-second chair stand test is a measure of leg strength and is measured by the number of times an individual can sit and stand from a chair in 30 s [38]. Cognitive changes were measured in verbal fluency, and cognitive function via the Mini-Mental State Examination [39]. All outcomes were measured pre- and post-intervention.

Two studies investigated equine-assisted services from a perspective other than the person living with dementia [29, 30]. Lassell et al. [30] investigated well-being, meaning through social connection and functions in daily life of people living with dementia from their carer’s perspectives, whilst Fields et al. [29] examined the safety, acceptability and QoL outcomes for participants from the perspective of providers of the Equine-Assisted Activities Program [29]. Care partners of people living with dementia participated in face-to-face semi-structured interviews within 1–2 weeks of the intervention and discussed outcomes of the intervention for themselves and for people living with dementia [30]. Field notes detailing observations of the date, time, setting, events/activities and reactions of the participants, were guided by Glesne [40] and triangulated with interview data [30]. Similarly, face-to-face semi-structured interviews were conducted immediately after the conclusion of the Equine-Assisted Activities Program to explore providers perspectives of the intervention on QoL of long-term care residents with dementia [29]. The qualitative process of coding the interview data was guided by Neergaard et al. [41].

### Summary of findings

All included studies found a favourable impact of participating in equine-assisted services. Studies confirmed that participating in equine-assisted services had positive effects on social [28, 30, 31], emotional [27, 28, 30, 31], and behavioural [27] outcomes for people living with dementia, as well as improved physical health [26]. Where the two interventions of riding and gardening were compared [31], participants expressed positive QoL indicators for the domains of apparent affect (interest and pleasure) and participation (engaged gaze, communication, and active participation) for both interventions. However, complex active participation was observed for adaptive riding but not for adaptive gardening [31]; that is, riding participants engaged in two or more activities simultaneously (such as riding *and* petting the horse) whilst gardening participants participated in only one activity at a time (such as planting *or* weeding) [31]. Moreover, there were increased observations of pleasure and active participation for riding compared to gardening [31]. Negative QoL indicators for apparent affect (sadness, depression, anger or agitation) were not observed amongst participants of both interventions [31].

Fields et al. [28] also found a difference in the QoL indicators when comparing participants who engaged in the equine-assisted intervention compared to those participating in other routine activities at the care facility; pleasure, engagement and active participation were observed more frequently for those interacting with horses. Therefore, when compared to participants engaged in routine activities in the long-term care facility, a higher proportion of participants involved in equine activities had engaged gaze, movement, conversation, participation, and less agitation, indicating that equine-assisted activities have a favourable impact on QoL indicators for people living with dementia compared to routine activities in long-term care facilities [28].

Equine-assisted services also elicited an improvement in balance and functional mobility post-intervention compared to pre-intervention [26], and a reduction in behavioural problems and disruptive behaviours compared to a comparison group participating in regular adult day service activities [27].

From the perspective of providers and carers, equine-assisted interventions contributed to positive QoL outcomes by improving well-being, functional abilities, social participation and relations, and communication of participants with dementia [29, 30]. Moreover, providers found the Equine-Assisted Activities Program to be safe and acceptable for people living with dementia [29].

## Discussion

This is the first review to synthesise the extent of research investigating the impact of equine-assisted services on the health and wellbeing of people living with dementia. Six sources were identified, and these sources investigated the impact of equine-assisted services on cognitive, emotional, and physical health, and behavioural, emotional, and overall wellbeing outcomes.

This review found that equine-assisted services had positive effects on social [28, 30, 31], emotional [27, 28, 30, 31], and behavioural [27] outcomes for people living with dementia, as well as physical health improvements [26]. This concurs with review literature synthesising the impact of animal-assisted interventions for people living with dementia [11, 42, 43] which have found similar effects [11, 42]. Similar to the current review, research to date has confirmed that animal-assisted interventions improve QoL [11, 42], social behaviours [11], and physical health and activity [11, 42] for people living with dementia. Animal-assisted interventions have also contributed to a significant reduction in neuropsychiatric symptoms for people living with dementia [11, 15], particularly agitation and aggression [11, 42] and depression [14]. Differing from the broader animal-assisted intervention literature, no study included within this review examined the impact of participation in equine-assisted services on depression and anxiety in people living with dementia; this is worthy of further investigation, especially considering that people living with dementia can exhibit symptoms of depression and anxiety, and pharmacological treatments can have limited impact while also producing unwanted side-effects [1].

A novel finding of this review is the positive impact of equine-assisted services on dementia care partners, who have described the intervention as a “meaningful shared activity” [30] which improved mood, provided a sense of accomplishment and purpose, improved well-being, and supplied “meaning through social connections” for both care partners and people living with dementia [30]. Additionally, this review adds evidence supporting the argument that equine-assisted services are appropriate and acceptable for people living with dementia and providers of care [29, 30]. Few studies of animal-assisted interventions and equine-assisted services have examined caregiver and provider perspectives, so this finding provides preliminary evidence of promising outcomes for caregivers and of the acceptability and appropriateness of equine-assisted services for people living with dementia from providers perspectives.

One qualitative study investigating the impact of participating in equine-assisted services for people living with dementia reported that caregivers noticed an improvement in functions of daily life for people living with dementia [30]; however, the sample size of this study was small (*n* = 5) and care should be taken interpreting this result. This is contrary to findings from a review which found that participating in animal-assisted interventions did not significantly improve independence in activities of daily living [14] for people living with dementia. Consequently, further research investigating how participating in equine-assisted services may impact functions of daily life is warranted, as the intervention may prove to improve activities of daily living.

One study examined cognitive function in people living with dementia pre-post equine-assisted therapy [26], using the Mini-Mental State Examination, and although the Mini-Mental State Examination score improved (indicating improvement in cognitive function), the change was minimal. This finding is similar to findings from reviews investigating the impact of AAI for people living with dementia, where significant differences in cognitive function between intervention and control groups has not been identified [14, 15]. Together findings suggest that animal-assisted interventions on the whole, do not have an impact on cognitive function of people living with dementia. Considering the lack of evidence of the impact of animal-assisted interventions on long term maintenance of cognitive function for people living with dementia, this suggests an opportunity for future research.

Whilst, in general, the evidence surrounding the impact of equine-assisted services on people living with dementia is limited, two reviews investigating the impact of equine-assisted services for non-dementia populations found that most or all studies reported social benefits [20], physical improvement [20] and psychological benefits [44]. Equine-assisted services was most promising for depression, and behavioural and social issues for children and adolescents [20, 44], and depression and anxiety for adults [44]. The current review confirms these findings as participants of included studies experienced physical improvements (balance and functional mobility) [26], psychological and emotional benefits (reduction in behaviour problems, improved mood and wellbeing) [27, 30, 31] and social benefits (participation and conversation) [28, 30, 31].

Although there are limited studies on the utilisation of equine-assisted services for people living with dementia, the mix of provider and care partner perspectives, alongside the outcomes for people living with dementia, establishes a good foundation for future studies investigating equine-assisted services for people living with dementia. As Hackshaw [45] recommends, small studies could be used to design confirmatory studies. In summary, further evidence is required to investigate the efficacy of equine-assisted services for people living with dementia [42, 43] with rigorous methodology and improved quality to enhance validity and reliability of results and ensure generalisability [10, 11].

### Limitations and future considerations

Almost all reviews of animal-assisted interventions for people living with dementia establish that this area of research has a limited number of studies [10, 14, 15, 43] and sample sizes of studies are small [10, 11, 20]. This review also found that the included studies had small sample sizes, which can compromise the internal and external validity of a study, affecting the conclusions we can draw from a study and limiting its generalisability [46]. Given the limited scope of EAS interventions considered, with three distinct types of EAS evaluated across the six studies, it is not possible to definitively conclude the specific benefits of a particular type of EAS. It will be important for future research to investigate different iterations of EAS and the distinct impact they may have on the health and well-being of people living with dementia, including those with different types of dementia, at different stages, and with varying life experiences with equines. Consequently, the conclusions drawn from studies identified from this review should be interpreted with caution [45]. Furthermore, the studies included in this review were almost all from the USA, with a predominantly Caucasian sample, again limiting the generalisability of the findings. In contrast, it is important to highlight that studies synthesised were of high methodological quality, and involved the use of robust validated measures, supporting the strength of the findings.

Four of the six publications were from researchers that have collaborated in this field of research [28–31], reducing the pool of independent research on equine-assisted services for people living with dementia. The absence of control or comparison groups in most of the included studies limits the ability to attribute the positive effects of the interventions on people living with dementia to the equine animal alone [44]; that is, to what extent were the positive effects of equine-assisted services on people living with dementia due to the horse or were there other factors that contributed to the positive effects of equine-assisted services, such as being outdoors or in the presence of a care partner?

Even though there have been no cost-effectiveness or cost-benefit analyses done on animal-assisted interventions or equine-assisted services [42] for people living with dementia, there are indications that it may entail high-costs, with the upkeep of animals and requirements for specialized training and staff [42]. Accessing equine-assisted services in Australia can cost anywhere from $190 to $210 per hour for an individual, although rebates may be available to help keep costs down [47–49]. Cost-effectiveness and cost-benefit analyses are important as they can help elucidate the cost of an intervention in relation to the benefits and health outcomes and guide resource allocation, health care and policy decisions and priorities [50, 51].

Equine based activities, particularly riding, are associated with well documented injury risks, as equines are large, powerful, sentient, prey- animals with herd instincts whose behaviour is not always predictable [52] and may lead to injury. Three of the review studies identified equine-related risks and provided detail about mitigation strategies utilized in their programs [26, 27, 29] while the remaining three mentioned ‘safety measures’ without providing detail. The omission of detailed discussions of risks related to equine assisted programs could suggest risk of reporting bias as well as a focus for further research.

Finally, follow-up of the effects of the interventions, although rare [18], would be beneficial to conduct in future studies to establish if there are long-term effects of the interventions and provide further information on the efficacy of equine-assisted services for people living with dementia.

Despite these limitations, this review indicates positive impacts of equine-assisted services for people living with dementia, its acceptability and appropriateness from provider and care partner perspectives, of which little is known, and adds to the body of evidence of the positive effects of animal-assisted interventions on the health and wellbeing of people living with dementia.

## Conclusions

This is the first review to the authors knowledge of the impact of equine-assisted services on people living with dementia.Findings from this review indicate that equine-assisted services show promise in improving neuropsychiatric symptoms of dementia and the QoL indicators of emotional, social and physical wellbeing. Additional robust studies are required to confirm specific health and wellbeing domains which may be favourably impacted.

## Data Availability

No datasets were generated or analysed during the current study.
